# Pharmacokinetic data on high dose baclofen administration in unhealthy alcohol user in the ICU

**DOI:** 10.1016/j.dib.2019.104231

**Published:** 2019-07-08

**Authors:** Mickael Vourc'h, Eric Dailly, Yannick Hourmant, Ronan Bellouard, Pierre-Joachim Mahe, Guillaume Deslandes, Matthieu Grégoire, Karim Asehnoune

**Affiliations:** a Departments of Anesthesiology and Surgical Intensive Care, Hôtel-Dieu, University Hospital of Nantes, Nantes, France; b Clinical Pharmacology Department, University Hospital of Nantes, Nantes, France

**Keywords:** Baclofen, Pharmacokinetic, Intensive care unit, Agitation, Alcohol use disorder

## Abstract

In the intensive care unit, alcohol intake above the NIAAA recommendations regardless of the existence of an Alcohol Use Disorder (AUD), was associated with an increased risk of death and longer time on ventilator. This rises the hypothesis that unhealthy alcohol use may lead to specific issues when weaning the mechanical ventilation (i.e. agitation or its related complications) regardless of AUD or withdrawal syndrome. Thus, we proposed to use baclofen off-label to avoid agitation. The data presented in this article is related to the research article entitled: “Pharmacokinetics and toxicity of high-dose baclofen in ICU patients” Vourc'h et al., 2019 Data provided in this submission includes 1) the detailed methods for baclofen assay by mass spectrometric detection, 2) the supplementary population pharmacokinetic analysis presenting observed concentration vs. population or individual predicted concentration (raw data of the latter is also available), and 3) the algorithm for the adaptation of baclofen daily doses according of the renal clearance to assess the risk of toxicity in critically ill patients.

**Table:** Specifications table

Subject area	Pharmacokinetic, unhealthy alcohol use, off-label use of baclofen, acute kidney injury, renal failure, alcohol use disorder, alcohol withdrawal syndrome
More specific subject area	Prevention of agitation in unhealthy alcohol user in the ICU
Type of data	Table, text and figures
How data was acquired	Liquid chromatography – tandem mass spectrometry assay 3200 QTRAP^®^ (SCIEX, Villebon-sur-Yvette, France).
Data format	Analyzed data
Experimental factors	**In 20 patients under mechanical ventilation in the intensive care unit, blood samples were taken following enteral administration of baclofen (feeding tube) and analyzed by chromatography to determine drug concentration over a 8-h period after the intake.**
Experimental features	Mass spectrometric detection was performed in positive ion mode using selected reactant monitoring
Data source location	Nantes University Hospital, Clinical pharmacology department, Nantes, FRANCE.
Data accessibility	**Pharmacokinetic data are hosted with the article. Additional** de-identified data collected for the study, including individual participant data will be made available to others. The study protocol, statistical analysis plan, ethics committee approval will be made available on reasonable request by addressing an e-mail to the corresponding author.
Related research article	Vourc'h, M., Dailly, E., Hourmant, Y., Bellouard, R., Mahe, P. J., Deslandes, G. et al. (2019). Pharmacokinetics and toxicity of high-dose baclofen in ICU patients. *Progress in Neuro-Psychopharmacology & Biological Psychiatry*. http://doi.org/10.1016/j.pnpbp.2019.02.016[1]

**Table:** 

**Value of the data** • Baclofen may be an option to prevent agitation in the ICU for unhealthy alcohol users, but no randomized study is available up to now. • The high variability of pharmacokinetic properties in the critically ill, notably when kidney injury occurs, urged us to assess the risk of toxicity in this population. • The proposed algorithm to adjust doses to renal clearance did not lead to overshoot the toxic plasma concentration of 1.1 mg/L [2] regardless of renal failure. • These results were used to design a multicenter randomized study to assess the relevance of baclofen vs. placebo to prevent agitation in unhealthy alcohol user with or without AUD in the ICU [3].

## 1 Data

**Fig. 1:**
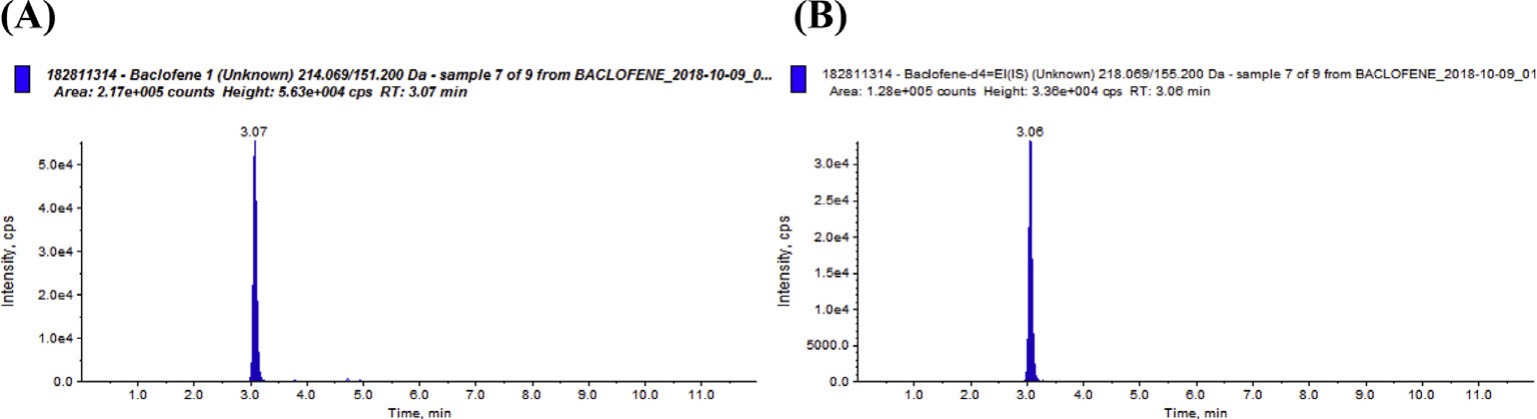
Chromatograms of a patient (A) plasma sample (0,17 mg/L) and the internal standard (B). These chromatograms with a high intensity signal for a low baclofen plasma concentration show the specificity and the high sensitivity of the assay. This assay is specific because no interfering chromatographic peak was found with a retention time close to the retention time of the baclofen peak (3.07 min). This assay is sensitive because the peak intensity (about 5.0 × 10^4^ cps) is highly superior to the background noise of the baseline although the baclofen plasma concentration (0,17 mg/l) is low.

**Fig. 2:**
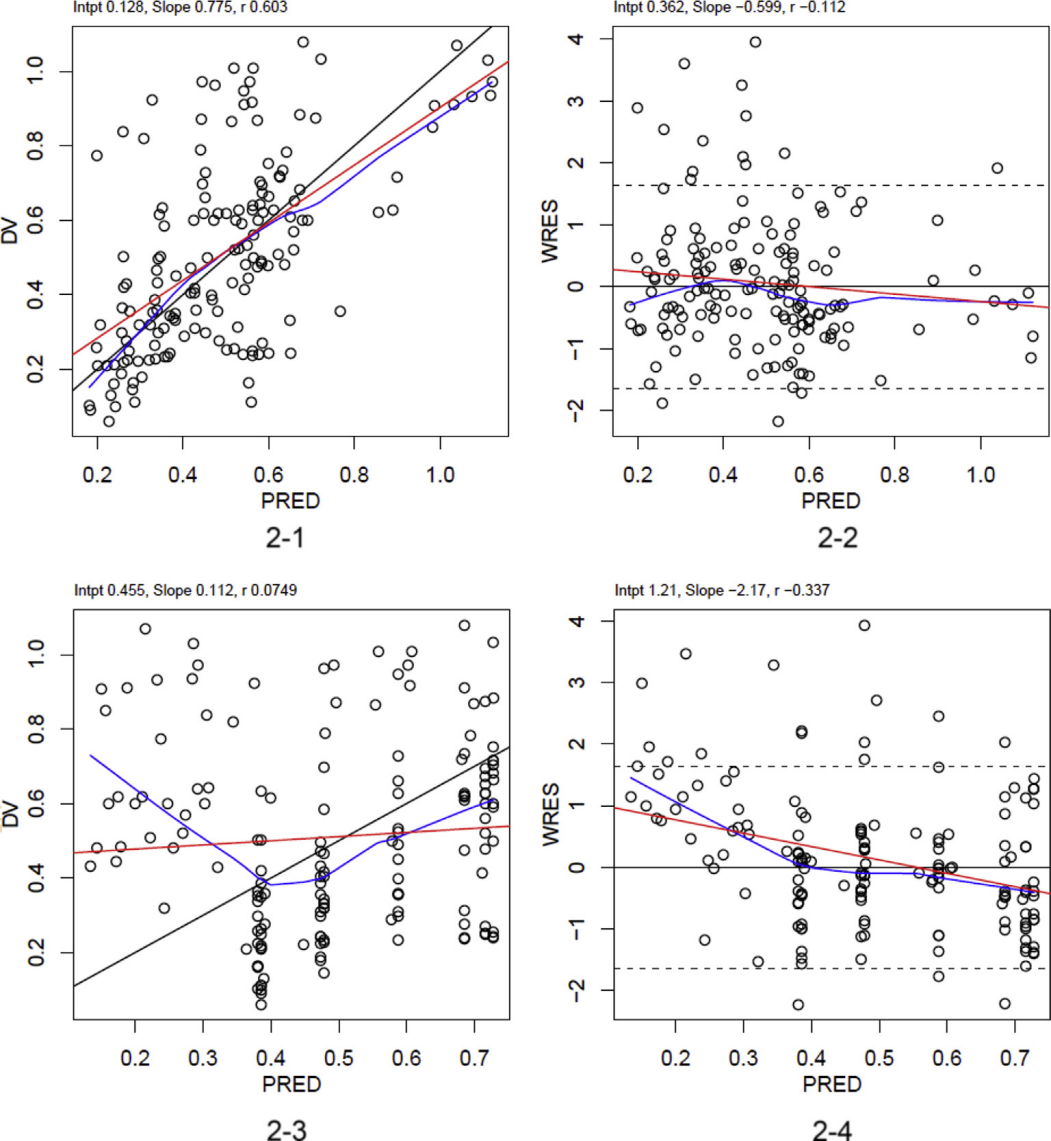
Population pharmacokinetic analysis of baclofen (color). Diagnostic plots for population pharmacokinetic analysis of baclofen: observed concentration [DV (mg/L)] versus population predicted concentration [PRED (mg/L)], weighted residuals (WRES) versus PRED in the final model (Figs. 2-1 and 2-2) and the base model (Figs. 2-3 and 2-4).

In a surgical intensive care, unhealthy alcohol users received off-label baclofen to prevent agitation during the awakening phase, trying to ease mechanical ventilation weaning. We created an algorithm for baclofen dose adaptation according to estimated glomerular filtration rate. The starting day of baclofen is referred as “day 1”: Patients received a loading dose of baclofen via an enteral feeding tube on day 1. Then daily doses were divided into 3 intakes until the weaning of mechanical ventilation. The pharmacokinetic study started on day 3: Samples were taken just before administration of baclofen and then from 30 min to 8 hours after administration (total of 8 samples). This article comprises: the method used to treat and analyze the samples (see **Methods for baclofen assay**), data files generated with the NONMEM software version 7.3 (Icon Development Solutions, Hanover, USA) based on baclofen assays (see a chromatogram example in Fig. 1 and additional results of the population pharmacokinetic analysis in Fig. 2. Finally, it includes the algorithm for baclofen doses adjustment according to the renal function (see Table 2).

## 2 Experimental design, materials, and methods

### 2.1 Methods for baclofen assay

Plasma samples were mixed with acetonitrile containing the internal standard (baclofen-d4) and centrifuged. The supernatant was transferred into glass tubes and then evaporated at 37 °C under a stream of nitrogen. Samples were recovered with mobile phase and then injected into the chromatographic system Agilent 1200 series (Agilent, Les Ulis, France). Separations were performed on an Aquasil^®^ 3 μm C18 50 mm × 2.1 mm column (Thermo Fisher Scientific, USA). The (MS/MS) system used was a 3200 QTRAP^®^ (SCIEX, Villebon-sur-Yvette, France). Mass spectrometric detection was performed in positive ion mode using selected reactant monitoring [baclofen m/z 214.07→151.2, baclofen-d4 (internal standard) m/z 218.07→155.2]. The method was found to be accurate (inaccuracy <10%) and showed good precision (imprecision <10%) The limit of quantitation is 0.01 mg/L. A chromatograph corresponding to a patient plasma sample is presented in Fig. 1.

### 2.2 Additional analysis for the pharmacokinetic model

The population pharmacokinetic analysis was performed using NONMEM software version 7.3 (Icon Development Solutions, Hanover, USA). The diagnostic plots corresponding to the final and base pharmacokinetic models are presented in Fig. 2. The raw data used to produce the graphs of the final model are presented in Table 1.

**Table 1:** Raw data to produce diagnostic plots of the final model.

PATIENT	DV	PRED	WRES	PATIENT	DV	PRED	WRES
1	0,1	0,24	−1,29	6	0,25	0,27	0,13
1	0,47	0,34	−0,06	6	0,35	0,37	0,33
1	0,91	0,54	2,17	6	0,48	0,57	−0,33
1	0,7	0,58	−0,29	6	0,48	0,45	0,09
1	0,56	0,56	−0,71	6	0,48	0,6	−0,68
1	0,36	0,43	0,29	6	0,4	0,47	−0,03
1	0,23	0,32	−0,15	6	0,31	0,36	−0,02
1	0,16	0,24	0,16	6	0,23	0,27	−0,33
2	0,26	0,33	−1,48	7	0,35	0,38	−0,34
2	0,42	0,43	−0,84	7	0,97	0,47	3,96
2	0,78	0,64	0,28	7	1,08	0,68	−0,94
2	0,88	0,67	1,55	7	1,04	0,72	1,38
2	0,72	0,62	−0,44	7	0,87	0,71	1,23
2	0,63	0,53	0,56	7	0,49	0,58	−1,7
2	0,47	0,42	0,68	7	0,39	0,47	−1,41
2	0,35	0,33	0,62	7	0,34	0,38	−0,5
3	0,21	0,2	−0,69	8	0,62	0,45	1,05
3	0,32	0,3	−0,68	8	0,62	0,48	0,61
3	0,62	0,5	1,06	8	0,64	0,56	0,1
3	0,59	0,54	0,04	8	0,64	0,58	−0,06
3	0,52	0,52	−0,85	8	0,6	0,58	−1
3	0,45	0,38	0,64	8	0,6	0,52	−0,4
3	0,36	0,28	0,92	8	0,6	0,47	0,28
3	0,26	0,2	0,48	8	0,6	0,43	0,95
4	0,42	0,26	0,42	9	0,64	0,35	2,36
4	0,5	0,46	−0,04	9	0,7	0,45	2,11
4	0,63	0,56	0,55	9	0,61	0,65	−0,82
4	0,66	0,6	−0,55	9	0,6	0,69	−0,64
4	0,67	0,59	−0,6	9	0,6	0,68	−0,27
4	0,73	0,45	2,77	9	0,53	0,55	−1,26
4	0,5	0,34	0,15	9	0,52	0,51	0,27
4	0,37	0,26	−0,67	9	0,5	0,35	−0,44
5	0,43	0,52	−0,11	10	0,22	0,24	0,12
5	0,44	0,55	−0,25	10	0,23	0,34	0,38
5	0,48	0,64	−0,81	10	0,24	0,54	−0,46
5	0,57	0,66	0,57	10	0,24	0,58	−1,4
5	0,52	0,66	−0,67	10	0,25	0,56	−1,62
5	0,51	0,62	−0,42	10	0,31	0,43	0,36
5	0,48	0,58	−0,24	10	0,22	0,3	−0,36
5	0,48	0,55	0,39	10	0,21	0,22	0,25

PATIENT	DV	PRED	WRES	PATIENT	DV	PRED	WRES

11	0,11	0,29	−1,04	16	0,78	0,2	2,9
11	0,33	0,38	−0,12	16	0,79	0,44	0,39
11	0,62	0,59	−0,4	16	0,87	0,51	0,86
11	0,72	0,63	1,3	16	0,92	0,56	0,48
11	0,63	0,61	0,35	16	0,95	0,54	0,63
11	0,36	0,48	−0,99	16	0,97	0,44	1,39
11	0,24	0,37	−0,3	16	0,92	0,33	1,87
11	0,17	0,28	0,2	16	0,84	0,26	1,6
12	0,11	0,56	−1,21	17	0,85	0,98	−0,51
12	0,33	0,65	−0,34	17	1,07	1,04	1,92
12	0,62	0,86	−0,68	17	1,03	1,11	−0,09
12	0,72	0,9	1,07	17	0,97	1,12	−0,79
12	0,63	0,89	0,1	17	0,94	1,12	−1,14
12	0,36	0,77	−1,51	17	0,93	1,07	−0,27
12	0,24	0,65	−0,75	17	0,91	1,03	−0,22
12	0,17	0,55	0,04	17	0,91	0,99	0,27
13	0,39	0,33	0,96	18	0,32	0,21	−0,68
13	0,41	0,43	−1,06	18	0,82	0,31	3,61
13	0,74	0,63	1,21	18	1,01	0,52	0,1
13	0,68	0,67	−0,31	18	1,01	0,56	0,24
13	0,65	0,66	−0,29	18	0,97	0,56	0,84
13	0,53	0,53	−0,09	18	0,87	0,44	3,26
13	0,41	0,42	−0,4	18	0,62	0,34	0,78
13	0,36	0,34	0,22	18	0,43	0,27	−0,77
14	0,22	0,26	−0,44	19	0,09	0,18	−0,58
14	0,24	0,36	0,25	19	0,15	0,28	−0,31
14	0,24	0,56	−0,52	19	0,28	0,48	0,08
14	0,24	0,6	−1,43	19	0,26	0,52	−1,29
14	0,27	0,59	−1,39	19	0,25	0,5	−1,31
14	0,3	0,45	−0,42	19	0,23	0,36	−0,02
14	0,3	0,34	0,5	19	0,19	0,26	0,54
14	0,28	0,27	0,78	19	0,1	0,18	−0,32
15	0,06	0,23	−1,56	20	0,5	0,26	2,55
15	0,32	0,32	1,74	20	0,59	0,36	0,55
15	0,32	0,53	−2,17	20	0,87	0,57	1,52
15	0,5	0,56	1,03	20	0,75	0,6	−0,52
15	0,42	0,54	−0,51	20	0,69	0,58	−0,61
15	0,29	0,4	−0,13	20	0,66	0,45	1,98
15	0,18	0,3	−0,47	20	0,43	0,34	−0,78
15	0,13	0,23	−0,08	20	0,3	0,26	−1,87

DV: observed concentration (mg/L), PRED: population predicted concentration (mg/L), WRES: weighted residuals.

These plots show an improved correlation between observed concentrations (DV) and predicted concentrations (PRED) obtained from the final model including the glomerular filtration rate in comparison with the results obtained from the base model without covariates: the equations established by linear regression between DV versus PRED (DV = 0.775 × PRED +0.128 in the final model versus DV = 0.112 × PRED+0.455 in the base model) and WRES (weighted residuals) versus PRED (WRES = −0.599 × PRED +0.362 in the final model versus WRES = −2.17 × PRED +1.21 in the base model) are respectively closer to the identity line (DV = PRED) and WRES = 0 in the final model (Figs. 2-1 and 2-2) than in the base model (Figs. 2-3 and 2-4).

### 2.3 Treatment adjustment in patients with renal failure

The following protocol of sedation was applied to adjust baclofen dose to renal function (see Table2).

**Table 2:** Protocol of sedation with baclofen dose adjustment according to renal function.

eGFR (ml/min/1.73m^2^)	Day 1, Baclofen loading dose (mg)	Day 2 and following days until extubation/tracheotomy (mg)
>90	150	50-50-50
89–60	100	30-20-50
30–59	70	20-20-30
<30 ou CVVHF^a^	50	20-10-20
IHD^b^	50	50 mg before each session
<15 without RRT^c^	50	No administration

eGFR: estimation glomerular filtration rate by the CKD-EPI equation. ^a^CVVHF: continuous veno-venous hemofiltration, ^b^IHD: intermittent hemodialysis, ^c^RRT: renal replacement therapy.
